# Women’s expectation of partner’s violence on HIV disclosure for prevention of mother to child transmission of HIV in North West Ethiopia

**DOI:** 10.1186/1756-0500-6-96

**Published:** 2013-03-14

**Authors:** Marelign Tilahun Malaju, Getu Degu Alene

**Affiliations:** 1 Department of Public Health, College of Medicine and Health Sciences, Arba-Minch University, P.O. Box 21, Arba-Minch, Ethiopia; 2 School of Public Health, College of Medicine and Health Sciences, University of Gondar, P.O. Box 196, Gondar, Ethiopia

**Keywords:** Male partner violence, PMTCT, Pregnant women

## Abstract

**Background:**

All violence against women has serious consequences for their mental, physical wellbeing, reproductive and sexual health including HIV infection and no study was conducted in this regard in Ethiopia and particularly in the present study area.

**Findings:**

A cross-sectional study was conducted in Gondar town from 22 July–18 August 2011*.* Of the 400 pregnant women who actively participated in this study, 314 (78.50%) expected a negative reaction for HIV positive test result from their partners. A positive reaction from the partner was associated with women having their own income (Adjusted odds ratio (AOR) (95% CI) =2.18 (1.21, 3.92)), residing in the urban areas (AOR (95% CI) =2.26 (1.21, 4.22)), having education level of secondary level and above (AOR (95% CI) = 6.05 (3.12, 11.72)), not having a stigmatizing attitude towards people living with HIV (AOR (95% CI) = 2.15 (1.24, 3.73)), having a positive attitude towards counselors (AOR (95% CI) = 2.46 (1.42, 4.25)) and being able to access health facilities (AOR (95% CI) = 2.35(1.22, 4.50)).

**Conclusion:**

Most of the participants in this study expected their partner to react negatively towards a positive HIV test result. Since women’s having their own income is strongly associated with a positive partner’s reaction on HIV test disclosure for prevention of mother to child transmission of HIV services, emphasis should be given for education and economic empowerment of women. A well functioning and accessible health facility with prevention of mother to child transmission of HIV service is important, especially in rural areas.

## Background

The HIV/AIDS pandemic remains a major public health challenge in sub-Saharan Africa. In 2011 the prevalence of HIV in Ethiopia was 1.5% with differentials: urban (4.2%), rural (0.6%), male (1.0%) and female (1.9%) [1]. The HIV/AIDS pandemic in sub-Saharan Africa has become a women’s health issue, with women accounting for 60% of people living with HIV [2]. HIV testing of men also remains challenging, with an estimated 6.1% of men in sub-Saharan Africa having ever been tested for HIV and receiving the results [3]. According to the Ethiopian Demographic and Health Survey (DHS) 2011, the prevalence and discordant rate of HIV among couples is 1.7% and 1.1% respectively [1].

Studies have shown that the utilization of prevention of mother to child transmission of HIV services by the pregnant women is influenced both by factors related to the health system such as accessibility of provider health initiative services and by individual factors such as fear of disclosure of HIV results, lack of male partner support, fear of domestic violence, abandonment and stigmatization [4-8]. Before the introduction of male involvement in the prevention of mother to child transmission of HIV programs, it was observed that a lot of pregnant women were shunning HIV testing because they had no consent from their husbands. Those who had courage to go for test, if tested positive were afraid to disclose their sero- status to their husbands because they thought their husbands would accuse them of infidelity or even face divorce. Some HIV positive women who had courage to inform their husbands, faced divorce, violence or accused of infidelity, some were not even allowed to continue with prevention of mother to child transmission of HIV interventions. This resulted in low uptake of prevention of mother to child transmission of HIV services by HIV positive women [9].

Domestic violence is common in Ethiopia, in both urban and rural families. According to the Ethiopian Demographic and Health Survey (DHS) 2011, 68 percent of women agree that wife beating is necessary. In rural areas 76 percent agree for wife beating, compared with 46 percent of urban women [1]. All violence against women has serious consequences for their mental, physical wellbeing, reproductive and sexual health including HIV infection as indicated in DHS 2011report that HIV prevalence is higher among women (1.9%) than men (1.0%). In Ethiopia only 8% of HIV infected pregnant women have received antiretroviral drugs to reduce the risk of mother to child transmission of HIV/AIDS during 2009 [10]. When a society tolerates and accepts violence against women, its eradication is more difficult. As part of the response to the problem, the Government of Ethiopia revised its family law in 2000 and its criminal law in 2005 to protect the rights of women and children and to promote gender equality and equity [1].

Since its introduction in sub Saharan Africa, male partner involvement has made an impact in reducing maternal to child transmission of HIV nevertheless it has its own challenges [11,12]. However, role of male partners in prevention of mother to child transmission of HIV programs and associated factors has not been well studied in Ethiopia and particularly in the present study area. Therefore, the objective this study is to assess women’s expectation of partner’s violence on HIV disclosure for prevention of mother to child transmission of HIV and associated factors which is preceded by two other studies aimed at assessing utilization of provider initiated HIV testing and counseling as an intervention for prevention of mother to child transmission of HIV and determinant factors of pregnant mothers knowledge on mother to child transmission of HIV and its prevention.

## Findings

Health institution based cross-sectional quantitative study was conducted in North West Ethiopia and the study period was from July 22 – August 18, 2011. The source population was all pregnant women attending antenatal care in public health facilities since these health facilities serve the majority of the population in antenatal care service especially the rural and poor population. Sample size was determined using the formula of a single population proportion estimation and calculated using software Epi-info stat calc. by taking 59% proportion, 5% of absolute precision and with 95% confidence interval. Non-response rate in this study was estimated to be 10% i.e. 38, and hence an overall sample size of 410 Pregnant women were recruited in the study. Stratified sampling technique was used to select the study units from five health centers and one hospital. Based on the number of customers who visited each health institution during the previous ten months, proportional allocation of the total sample size was carried out to attain the required sample size in each health institution. Finally, the determined sample for each health institution was achieved through exit interview from systematically sampled and voluntarily consenting pregnant women. The interviewers were clinic staffs providing antenatal care services and they were trained on the data collection and interview techniques. A structured questionnaire was used to elicit the following information from the study participants: socio-demographic data, male partner’s reaction for HIV positive result, number of antenatal care visits, comprehensive knowledge on HIV, knowledge on prevention of mother to child transmission of HIV, accessibility of health facility and stigmatizing attitude towards people having HIV.

Expected positive partners reaction was defined to be women’s expectation of their partner’s reaction on HIV disclosure and giving any of the following responses: accept me without complain, emotional support or strengthen relationship.

The completeness and consistency of data was established through direct and daily supervision by the supervisor and principal investigator. Data coding, cleaning and verification were performed to assure quality of data. Data were entered and analyzed using SPSS software version 16. Descriptive statistics such as frequencies and proportion was used to describe the study population in relation to relevant variables. Explanatory variables found to be statistically significant in bivariate logistic regression analysis were entered into multiple logistic regression analysis (backward stepwise method) for adjustment of confounders. Odds ratio, confidence interval and P-value were computed to assess the presence and degree of association between dependent and independent variables. Ethical clearance to conduct the study was obtained from Ethical Review Board, School of public health, University of Gondar and permission to conduct the study in each health facilities was secured from the respective Health institutions in Gondar Town. Verbal informed consent from each study participants was obtained after clear explanation about the purpose of the study.

### Socio-demographic characteristics of the study participants

Out of the total 400 pregnant women who participated in this study, about 285 (71.2%) of them were from urban areas while the rest 115 (28.8%) were from rural part of the study area.

Three hundred sixty one (90.2%) of the respondents were married followed by those who were divorced/separated/widowed 20 (5.0%) and the majority 194 (48.5%) of them had no education, followed by those who attended school secondary and above education 168 (42.0%).

The most frequent occupation was housewife (59.0%) seconded by government employed (16.0%) and merchant (10.2%), respectively. Majority 391 (97.8%) of them were Amhara by ethnicity followed by Tigre and 361 (90.0%) were followers of orthodox Christianity followed by Muslim 35(8.8%). Most of the study participants (46.0%) were in the age range between 25–34 years with mean (±SD) age of 25.4 (±5.3) and majority (73.2%) of them do not have their own income (Table 1).

**Table 1:** Socio-demographic characteristics of pregnant women attending ANC in health facilities of Gondar town, North West Ethiopia, 2011

**Variables**	**Urban**	**Rural**	**Total**
	**n (%)**	**n (%)**	**n (%)**
**Age **[Mean(±SD) = 25.4 (±5.3)]			
15–24	141(35.2)	37 (9.3)	178 (44.5)
25–34	126 (31.5)	58 (14.5)	184 (46.0)
35-49	18 (4.5)	20 (5.0)	38 (9.5)
**Ethnic group**			
Amhara	279 (69.8)	112 (28.0)	391(97.8)
Tigray	5 (1.2)	3 (0.8)	8 (2.0)
Gurage	1 (0.2)	0 (0.0)	1 (0.2)
**Religion**			
Orthodox Christian	249 (62.2)	111 (27.8)	360 (90.0)
Muslim	31 (7.8)	4 (1.0)	35 (8.8)
Protestant	5 (1.2)	0 (0.0)	5 (1.2)
**Education**			
No education	108 (27.0)	86 (21.5)	194 (48.5)
Primary Education	28 (7.0)	10 (2.5)	38 (9.5)
Secondary & above	149 (37.2)	19 (4.8)	168 (42.0)
**Occupation**			
Government employed	57 (14.2)	7 (1.8)	64 (16.0)
Merchant	39 (9.8)	2 (0.5)	41 (10.2)
House wife	139 (34.8)	97 (24.2)	236 (59.0)
Student	35 (8.8)	2 (0.5)	37 (9.2)
Others*	15 (3.8)	7 (1.8)	22 (5.5)
**Marital Status**			
Never married	17 (4.2)	2 (0.5)	19 (4.8)
Married/living together	255 (63.8)	106 (26.5)	361 (90.2)
Divorced/separated/widowed	13 (3.2)	7 (1.8)	20 (5.0)
**Women having their own income**			
Yes	55 (13.8)	52 (13.0)	107 (26.8)
No	230 (57.5)	63 (15.8)	293 (73.2)

* Bartender, daily laborer and Jobless.

### Expected partner’s violence on pregnant women towards HIV test disclosure for prevention of mother to child transmission of HIV

The results in Table 2 indicate that larger proportion of women (78.5%) tend to expect negative reaction from their partners on HIV positive test result for prevent mother to child transmission of HIV and only 21.5% of the study participants expect positive reactions from their partners if they are found to be HIV positive for prevent mother to child transmission of HIV.

**Table 2:** Expected partners’ reaction for HIV positive test result among pregnant women attending antenatal care in health facilities of Gondar town, North West Ethiopia, 2011

**Expected partner’s reaction on HIV test disclosure**	**n = 400**
Any negative reaction	314 (78.5%)
Insult me	51 (12.8%)
Psychological harassment	38 (9.5%)
Physical violence	27 (6.8%) 90
Marriage disruption	90 (22.5%)
Stop financial support	108 (27.0%)
Any positive reaction	86 (21.5%)
Accept me without complain	45 (11.3%)
Emotional support	17 (4.3%)
Strengthen relationship	24 (6.0%)

As to the specific partner’s reaction, most of the study participants expect termination of financial support from their partner (27.0%) and marriage disruption (22.5%) but relatively a small proportion of women expect positive responses from their partner (11.3%) of them said that he would accept me without any negative complain and 6.0% of them expect strengthen relationship (Table 2).

### Factors associated with expected male partners’ violence on pregnant women towards HIV test disclosure for prevention of mother to child transmission of HIV

In order to measure the association of expected male partners’ violence on pregnant women towards HIV test disclosure for prevention of mother to child transmission of HIV with a number of explanatory variables, crude OR and adjusted OR with 95% CI were employed. After controlling for confounders, the association between selected explanatory variables and male partners’ violence on pregnant women towards prevention of mother to child transmission of HIV is presented in Table 3.

**Table 3:** Association of male partner’s reaction towards HIV test disclosure for PMTCT of HIV with each explanatory variable (Crude & adjusted OR), Gondar, North West Ethiopia, 2011

**Explanatory Variable**	**Partner’s reaction on HIV test disclosure for prevention of mother to child transmission of HIV**		**Crude OR (95% CI)**	**Adjusted OR (95% CI)**	**P-value**
	**Positive(1)**	**Negative(0)**			
**Women having their own income**					
Yes	58	49	3.84 (2.41, 6.13)	2.18 (1.21, 3.92)	0.009
No	69	224	1.00	1.00	
**Residence**					
Urban	227	58	5.99 (3.66, 9.41)	2.26 (1.21, 4.22)	0.011
Rural	46	69	1.00	1.00	
**Education level**					
No Education	93	101	1.00	1.00	<0.001
Primary education	27	11	4.16 (1.73, 10.01)	2.44 (0.92, 6.49)	0.073
Secondary & above	153	15	11.08 (6.08, 20.19)	6.05 (3.12, 11.72)	<0.001
**Type of health facility**					
Hospital	75	24	1.00	1.00	0.063
Health center	198	103	1.63 (.97, 2.73) 1.00	1.89 (0.97, 3.69)	
**Holding stigmatizing attitude towards people living with HIV**					
Yes	93	90	1.00	1.00	0.006
No	180	37	4.71 (2.98, 7.44)	2.15 (1.24, 3.73)	
**Attitude towards counselors**					
Positive	192	51	3.53 (2.28, 5.48)	2.46 (1.42, 4.25)	0.001
Negative	81	76	1.00	1.00	
**Accessibility of Health facility**					
Yes	237	68	5.71 (3.48, 9.37)	2.35 (1.22, 4.50)	0.010
No	36	59	1.00	1.00	

**Adjusted for:** frequency of antenatal care visits, comprehensive knowledge on HIV, accessibility of health facility, holding stigmatizing attitude towards people having HIV, Women having their own income, residence, education level, attitude towards counselors and type of health facility.

For explanatory variables having more than two categories, the overall significance is given by their corresponding P-values.

The assessment made whether the required assumptions for the application of multiple logistic regression was fulfilled showed that this parsimonious model adequately fits the data as P = 0.458 (by using Hosmer and Lemeshow test).

Compared to women who live in the rural areas, those women living in the urban areas were about 2.26 times [OR & (95% CI) = 2.259 (1.210, 4.217)] more likely to expect positive partners reaction on HIV disclosure for prevention of mother to child transmission of HIV in Gondar town, North West Ethiopia.

Women with education level of secondary and above were 6.05 times [OR & (95% CI) = 6.048 (3.121, 11.720)] more likely to expect positive partners reaction on HIV disclosure for prevention of mother to child transmission of HIV than those with no education.

Women who have their own income were 2.18 times [OR & (95% CI) = 2.180 (1.213, 3.920)] more likely to expect positive partners reaction on HIV disclosure for prevention of mother to child transmission of HIV than those who do not have their own income.

Women who do not have stigmatizing attitude towards people living with HIV were 2.15 times [OR & (95% CI) = 2.149 (1.240, 3.725)] more likely to expect positive partners reaction on HIV disclosure for prevention of mother to child transmission of HIV than those who have.

Compared to women who cannot access health facility (with in 5 km of distance) those who can access were about 2.35 times [OR & (95% CI) = 2.345 (1.223, 4.498)] more likely to expect positive partners reaction on HIV disclosure for prevention of mother to child transmission of HIV. Finally, women who have positive attitude towards counselors were 2.46 times [OR & (95% CI) = 2.455 (1.419, 4.246)] more likely to expect positive partners reaction on HIV disclosure for prevention of mother to child transmission of HIV than those who have negative attitude.

## Discussion

The current study focuses on women’s expectation of male partner’s violence on pregnant mothers towards HIV disclosure for prevention of mother to child transmission of HIV in Gondar town, North West Ethiopia. Three hundred fourteen (78.5%) mothers in this study expected negative reactions from their partners and only eighty six (21.5%) of them expect positive reactions from their partner if they are found to be HIV positive for prevention of mother to child transmission of HIV. This finding is different from the results obtained from one urban hospital in south west Ethiopia where 65.1% and 18.1% of them expected positive and negative reaction from their partner respectively. This might be due to the fact that our study is conducted in six health centers and one hospital where majority of the participants in this study were from rural setting indicating that difference of male partner’s reaction in rural and urban setting [13]. So far, the Government of Ethiopia revised its family law in 2000 and its criminal law in 2005 to protect the rights of women and children and to promote gender equality and equity [1].

As to the specific partner’s reaction, majority (27.0% and 22.0%) of them said that he will stop financial support and marriage disruption respectively and a small proportion (11.3% and 4.3%) of them said that he will accept me without complain and give emotional support respectively and this suggests a need to promote couple counseling and testing in the antenatal care clinics as recently shown in Uganda [14]. This finding is also in line with the findings from two rural districts of Zimbabwe [15].

Positive association was reported between expected positive partner’s reaction on HIV disclosure for prevention of mother to child transmission of HIV and women having their own income on the other hand and this could be that women with lower income are less able to make decisions on their own as they are less empowered economically and this is in line with the finding from Tanzania [16].

Accessibility of health facility (with in 5 km distance) was positively associated with expected positive partner’s reaction on HIV disclosure for prevention of mother to child transmission of HIV. The possible explanation for this association could be that the less a health facility is far away from the woman’s house the more a pregnant woman comes in contact with the health center with her partner and the more likely her partner is to hear about prevention of mother to child transmission of HIV, among other preventive messages and services and the more likely he react positively. Improving accessibility of health facilities with prevention of mother to child transmission services is therefore a high priority for improving prevention of mother to child transmission of HIV as shown in Thailand [17].

Women in urban areas were also found to expect positive partner’s reaction on HIV disclosure for prevention of mother to child transmission of HIV than those who reside in rural areas and this could be that partners’ reaction in the rural area is different from urban areas and this indicates the need to exert efforts more in the rural areas where accessibility of health facilities and prevention of mother to child transmission of HIV messages are not well addressed.

Regarding association of education level of pregnant women with expected partner’s reaction in this study, those women with secondary and above education level were more likely to expect positive partner’s reaction on HIV disclosure for prevention of mother to child transmission of HIV than those with no education and this might be due to the fact that uneducated women might be different from educated once in their decision making ability and discussion with their partner in prevention of mother to child transmission of HIV. This possible explanation is also in line with the finding from Burkina Faso where women having secondary and above education level were found to be less fearful to disclose their HIV status to their partner than those who are illiterate [18].

### Limitations

Being a cross-sectional survey, causality cannot be inferred from these findings. The study is limited by being facility based and therefore precludes generalization to all pregnant women in Ethiopia indicating a need for further study of male partner’s influence and involvement in prevention of mother to child transmission of HIV using a more representative sample of pregnant women in the country. Despite this limitation, the study provides useful information that will inform health service planners to design a strategy to improve male partner’s awareness and positive influence on prevention of mother to child transmission of HIV programs in Ethiopia.

## Conclusion

Most of the study participants in this study anticipate negative partner’s reaction for prevention of mother to child transmission of HIV. Majority of them expect termination of financial support and marriage disruption from their partner if they are found to be HIV positive for prevention of mother to child transmission of HIV. Mothers having their own income, having education level of secondary and above and having accessible health facility were found to be less influenced negatively by their partners. This indicates, therefore, the need to give more emphasis for education and economic empowerment of women and there should be well functioning and accessible health facilities with prevention of mother to child transmission service in the country especially in the rural areas.

## Competing interests

The authors declare that they have no competing interests.

## Authors' contributions

MT was investigator, involved in proposal writing, designing, and recruitment and training of supervisors and data collectors, analysis and write-up and in all stages of the project implementation. He did most of the analysis and write up of the paper. GD contributed in the designing of the methodology, lead investigator and involved in designing of project proposal, design of questionnaires, supervision and involved in the analysis stage of the project and final approval of the paper. All authors read and approved the final manuscript.
